# Association Between Types of Posterior Staphyloma and Refractive Error After Cataract Surgery for High Myopia

**DOI:** 10.3389/fneur.2021.736404

**Published:** 2021-11-30

**Authors:** Jing Wu, Ruochen Wang, Can Liu, Yu Zhou, Ziyuan Jiang, Fang Liu

**Affiliations:** ^1^Department of Ophthalmology, Tenth People's Hospital Affiliated of Tongji University, Shanghai, China; ^2^Department of Ophthalmology, Tenth People's Hospital Chongmin Branch, Shanghai, China; ^3^Department of Epidemiology, University of Michigan School of Public Health, Ann Arbor, MI, United States

**Keywords:** high myopia, refractive error, posterior staphyloma, cataract surgery, biomarker

## Abstract

**Purpose:** To investigate the association between different types of posterior staphyloma (PS) and refractive error (RE) after cataract surgery in patients with high myopia.

**Methods:** This retrospective study included 113 eyes of 113 highly myopic patients with PS. PS was detected using a wide-field fundus imaging system. PS was classified into wide macular, narrow macular, and other types. RE equaled the actual spherical equivalent (SE) minus the targeted SE values 3 months after cataract surgery.

**Results:** The rates of wide macular, narrow macular, and other types of PS were 46.02, 39.82, and 14.16%, respectively. There were no significant differences in best corrected distance visual acuity (BCDVA) or SE among the three classifications of PS before cataract surgery (*P* > 0.05). However, postoperative BCDVA and SE were significantly different among the three types of PS patients (*P* < 0.05). The average RE values were 0.98 ± 1.00 D, 0.19 ± 0.87 D, 0.13 ± 0.59 D, respectively; the statistical differences of RE were <0.01, <0.01, and 0.81 (wide macular vs. narrow macular, wide macular vs. other types, narrow macular vs. other types), respectively. Multivariate linear regression analysis revealed that higher hyperopia RE after surgery was associated with wide macular staphyloma (*P* < 0.001), more myopic SE (*P* = 0.003), and increased BCDVA (*P* = 0.002) before surgery.

**Conclusions:** Wide macular PS may be associated with more hyperopic RE; it may serve as a critical biomarker of hyperopic RE after cataract surgery in highly myopic patients.

## Introduction

High myopia is considered a neurovascular disease and may affect both the retinal microvascular network and optic nerve, causing visual impairment (1–4). The prevalence of high myopia is increasing worldwide, and remains the leading cause of irreversible vision loss in adults due to associated complications (5, 6). Posterior staphyloma (PS) is a hallmark of myopic retinopathy (7, 8), which was defined by Spaide in 2013 as “an outpouching of the wall of the eye that has a radius of curvature that is less than the surrounding curvature of the wall of the eye” (9). PS is present in about one in three to one in two of highly myopic eyes in adults, and the prevalence of PS is associated with increasing age and longer axial length (10–12).

In 1977, Curtin first classified PS into 10 types according to its morphological characteristics on fundoscopic examination (13); this classification is still used in clinical practice. Recently, PS has become more easily detected using new instruments such as optical coherence tomography and high-resolution, three-dimensional magnetic resonance imaging (3D-MRI) (14, 15). However, traditional fundus images often cannot include the entire border of the PS, in particular, in wide macular staphyloma; in addition, screening with 3D-MRI may not be feasible in the clinic. In 2014, Ohno-Matsui analyzed the morphological characteristics of the whole eye by applying wide-field fundus imaging, so that even a large field of PS could be imaged directly and comprehensively. More importantly, this method simplified the classification of PS according to its location and distribution, in contrast to traditional classification (10, 16).

Complicated cataract is a common complication in patients with high myopia (17); however, precise measurement of intraocular lens (IOL) power in highly myopic eyes with cataract remains a challenge. It is known that greater axial length (> 26 mm) affects the precision of the IOL power estimates in highly myopic eyes (18). As PS is accompanied by axial length elongation and morphological changes to the eyeball of highly myopic eyes, PS is classified into different types based on location and distribution. However, few previous studies have focused on whether different types of PS may affect postoperative refractive errors (RE) in highly myopic eyes. Thus, the purpose of this study was to examine the associations between different types of PS and RE after cataract surgery in patients with high myopia, using wide-field fundus imaging technology.

## Materials and Methods

This study retrospectively reviewed preoperative medical records of 113 eyes of 113 highly myopic patients with PS (spherical equivalent values of more than −6.00 D or axial length of >26 mm). For patients with both eyes involved, only data from the right eye were used for statistical analyses. All patients underwent cataract surgery between January 1, 2018, and July 31, 2020, at the cataract clinic of the Shanghai Tenth People's Hospital affiliated with Tongji University. Cataract was graded at the slit lamp according to the Lens Opacification Classification System (LOCS) lll classification (1) as follows: nuclear opalescence (NO); nuclear color (NC); cortical cataract (C) and posterior subcapsular cataract (P). This study was approved by the Clinical Research Ethical Committee of the Shanghai Tenth People's Hospital affiliated with Tongji University and adhered to the principles of the Declaration of Helsinki (clinical study registered at www.chictr.org.cn, accession number ChiCTR2000036875). Written informed consent was provided for the use of the participants' medical data for clinical research purposes. The ocular exclusion criteria were as follows: (a) irregular corneal astigmatism, glaucoma, uveitis, retinal detachment, and the presence of a full-thickness macular hole; (b) history of eye trauma or previous ocular surgery that may affect ocular morphology; (c) serious myopic retinopathy such as choroidal neovascularization (CNV), macular epiretinal membranes, macular retinoschisis and so on; (d) cataract which exceeded N5, C4, and P4.

All participants underwent a complete preoperative examination. Baseline characteristics of patients with highly myopic eyes with PS were recorded, including age, sex, best corrected distance visual acuity (BCDVA), and spherical equivalent (SE). Keratometry (K) measurements were performed using an anterior segment swept-source optical coherence tomography SS-1000 (Tomey Corporation, Nagoya, Japan). Preoperative IOL calculations and axial lengths were measured using an IOLMaster 700 (Carl Zeiss, Meditec AG, Germany). The actual refractive outcomes of the participants were recorded 3 months after the surgery. RE was calculated based on the achieved postoperative SE minus the targeted SE.

### Surgical Technique for IOL Implantation

All cataract surgeries were performed by a single experienced surgeon. In highly myopic patients with complicated cataracts, lens aspiration followed by implantation of a foldable posterior chamber IOL (ZCB00, Abbott Medical Optics, Santa Ana, CA, USA) into the capsular bag was performed. The IOL power was calculated based on the Barrett Universal II formulas. The predicted refractive outcomes targeted approaching −3 (D) under correction.

### Classification of Posterior Staphyloma

PS was determined and classified using wide-field fundus imaging of 200° of the retina, based on Ohno-Matsui K's definition (10). After dilution, fundus imaging was performed using an Optos 200Tx scanning laser ophthalmoscope (Optos PLC, Dunfermline, UK). Two experienced doctors determined the types of PS, which included wide macular staphyloma, narrow macular staphyloma, and other types of staphylomas, based on location and distribution. Wide macular staphylomas were defined as the nasal edge of the macular staphyloma placed more nasally beyond the nasal edge of the optic disc. While the nasal edge of the macular staphyloma was along the nasal edge of the optic disc, the eyes were considered to be narrow macular staphyloma.

**Figure 1:**
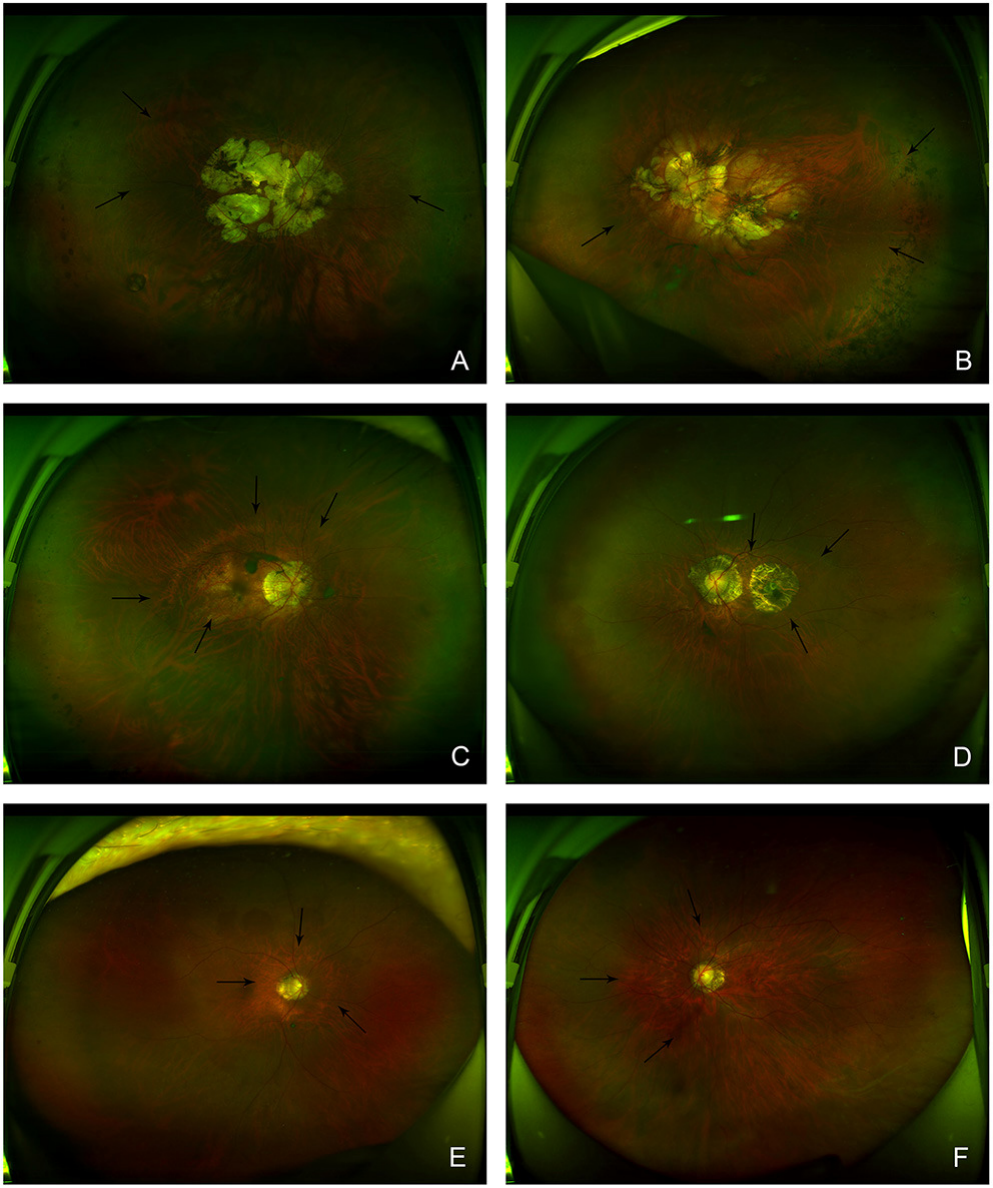
Representative wide-field fundus images of posterior staphyloma. **(A,B)** Eyes with a wide macular staphyloma. Posterior fundus shows fibrovascular tissue and focal chorioretinal atrophy. Focal chorioretinal atrophy has spread widely and the posterior fundus shows a bare sclera appearance. **(C,D)** Eyes with a narrow macular staphyloma. Posterior fundus shows a relatively narrower focal chorioretinal atrophy. **(E,F)** Eyes with other types of staphylomas. In a typical peripapillary staphyloma, focal chorioretinal atrophy is closer-set and tends to be restricted to the optic disc. Arrows shows the edge of posterior staphyloma.

The study slightly modified the classification of PS described by Ohno-Matsui because wide macular staphyloma and narrow macular staphyloma are the most common types of PS, according to our clinical observation and previous studies (12); the remaining PS types (including inferior staphyloma, peripapillary staphyloma, nasal staphyloma, and types not otherwise specified by the Ohno-Matsui classification) were grouped as the “other” type of PS in this study. Figure 1 shows representative wide-field fundus images of the different types of PS. We repeated three times of the measurements by the experienced physicians to make sure the reliability of our results.

### Statistical Analyses

SPSS version 22.0 software (Chicago, IL, USA) was used for statistical analyses. Data were presented as the mean ± standard deviation. BCDVA was presented as the logarithm of the minimum angle of resolution. One-way analysis of variance (ANOVA) followed by a *post-hoc* test was used to compare the results of different types of PS. Univariate and multivariate analyses using linear regression models were performed to analyze the impact of various variables on RE. Statistical significance was set at *P* < 0.05.

## Results

Among 113 patients, 64 (56.64%) patients were female. A total of 52 (46.02%), 45 (39.82%), and 16 (14.16%) eyes had wide macular, narrow macular and other types of staphylomas respectively. And other types of staphylomas included 10 (8.85%), 4 (3.54%), and 2 (1.77%) eyes with the peripapillary, inferior, and other staphyloma types, respectively. There was no significant difference in age, axial length, Km, BCDVA, or SE among the groups before cataract surgery (all *P* > 0.05). The patients' preoperative baseline characteristics are presented in Table 1.

**Table 1:** Preoperative baseline characteristics of patients with highly myopic cataract eyes and posterior staphyloma.

	**All**	**Wide, macular**	**Narrow, macular**	**Other types**	***P*-value**
No. of eyes (%)	113	52 (46.02%)	45 (39.82%)	16 (14.16%)	–
Age, year	64.73 ± 7.47	65.92 ± 7.14	64.00 ± 7.56	62.94 ± 8.14	0.27
Gender (male/female)	49/64	22/30	20/25	7/9	0.77
ACD, mm	3.47 ± 0.46	3.50 ± 0.41	3.47 ± 0.55	3.35 ± 0.34	0.52
AL, mm	28.78 ± 1.69	29.04 ± 2.03	28.60 ± 1.41	28.44 ± 1.03	0.32
Km, D	44.00 ± 1.28	43.94 ± 1.20	44.12 ± 1.39	43.85 ± 1.26	0.69
BCDVA, logMAR	0.52 ± 0.32	0.59 ± 0.27	0.46 ± 0.37	0.44 ± 0.27	0.06
SE, D	−11.49 ± 3.35	−11.52 ± 3.76	−11.49 ± 2.99	−11.42 ± 3.08	0.99

Data are shown as mean ± standard deviation. *Statistically significant at p <0.05. ACD, anterior chamber depth; AL, axial length; BCDVA, best corrected distance visual acuity; D, diopter; Km, mean keratometry; Log MAR, logarithm of the minimum angle of resolution; SE, spherical equivalent.

### Postoperative Refractive Outcomes of Highly Myopic Eyes With PS

Three months after cataract surgery in 113 highly myopic eyes with PS, postoperative refractive outcomes including BCDVA, actual SE, targeted SE, and RE were compared among the three subclassifications (Table 2). Postoperative BCDVA was better in eyes with narrow macula and other types of PS than in eyes with wide macular staphyloma (*P* = 0.01, 0.02, respectively). Among the three categories, the actual SE values were −2.00 ± 1.17 D, −2.92 ± 0.61 D, −2.90 ± 0.61 D in wide macular, narrow macular, and other types of PS groups, respectively. RE presented a hyperopic trend after IOL implantation, with a much greater deviation from the targeted refractive outcome in the macular wide staphyloma group than in the other two groups (*P* < 0.01, *P* = 0.01, respectively).

**Table 2:** Postoperative refractive characteristics of patients with highly myopic eyes and posterior staphyloma.

	**Posterior staphyloma**			***P*** **-value**		
	**Wide, macular**	**Narrow, macular**	**Other types**	**Wide, macular vs. Narrow, macular**	**Wide, macular vs. Other types**	**Narrow, macular vs. Other types**
BCDVA, logMAR	0.29 ± 0.39	0.14 ± 0.14	0.11 ± 0.10	0.01^*^	0.02^*^	0.66
Targeted SE, D	−2.97 ± 0.46	−3.11 ± 0.53	−3.02 ± 0.47	0.16	0.70	0.55
Actual SE, D	−2.00 ± 1.17	−2.92 ± 1.19	−2.90 ± 0.61	<0.01^*^	0.01^*^	0.63
RE, D	0.98 ± 1.00	0.19 ± 0.87	0.13 ± 0.59	<0.01^*^	<0.01^*^	0.81

*Data are shown as mean ± standard deviation*.

* *Significant at p <0.05. BCDVA, best corrected distance visual acuity; D, diopter; Log MAR, logarithm of the minimum angle of resolution; SE, spherical equivalent; RE, refractive error. RE equals the measured postoperative actual SE minus the targeted SE*.

### Refractive Error in Different Types of Posterior Staphyloma

**Figure 2:**
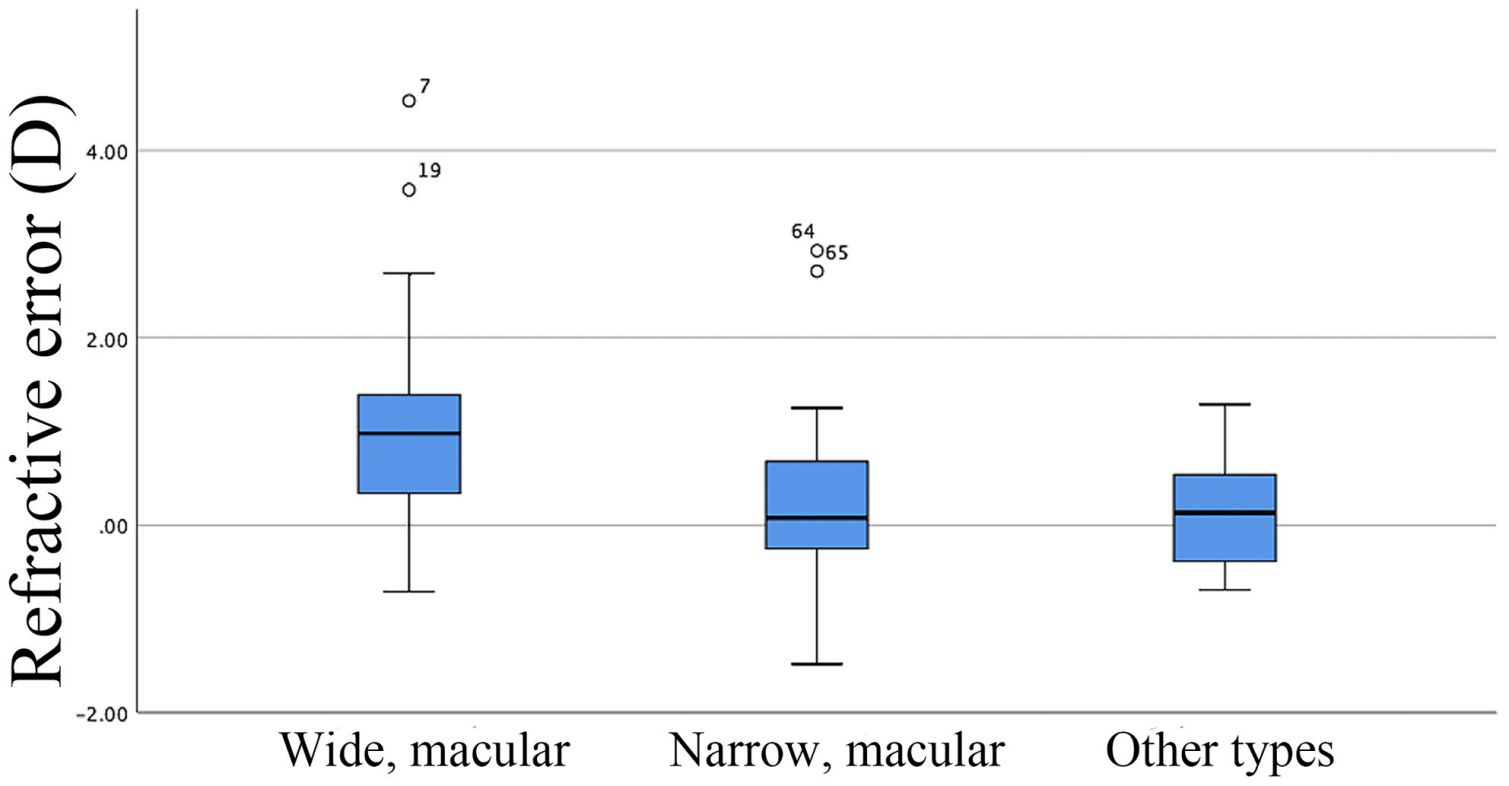
Distribution of postoperative refractive errors in different posterior staphyloma groups.

The distributions of RE in different types of PS after cataract surgery are shown in Figure 2. There was a hyperopic trend in highly myopic patients after IOL implantation. The RE (mean ± SD) values of the wide macular, narrow macular, and other types of PS groups were 0.98 ± 1.00 D, 0.19 ± 0.87 D, 0.13 ± 0.59 D, respectively, indicating statistically significant differences between wide macular vs. narrow macular (*P* < 0.01) and wide macular vs. other types of PS (*P* < 0.01). However, there was no difference between the narrow macula and other types of PS in this parameter (*P* = 0.81).

### Risk Factors Associated With Refractive Error in Highly Myopic Eyes With PS

Univariate and multivariate linear regression analyses were used to examine preoperative risk factors associated with RE after cataract surgery, including the types of PS, age, anterior chamber depth, Km, axial length, preoperative SE, and BCDVA (Table 3). The presence of wide macular staphyloma (*P* < 0.001), higher preoperative myopic SE (*P* = 0.003), and worse preoperative BCDVA (*P* = 0.002) were positively associated with higher hyperopic RE in the multivariate analyses.

**Table 3:** Univariate and multivariate analyses of risk factors for postoperative refractive errors.

**Variables**	**Univariate**			**Multivariate**		
	**β**	**95%CI**	***P***	**β**	**95%CI**	***P***
**PS**						
Others	Reference	Reference	–	Reference	Reference	-
Wide, macular	0.792	0.428, 1.157	<0.001^*^	0.726	0.330, 1.041	<0.001^*^
Narrow, macular	−0.065	−0.586, 0.456	0.805	−0.010	−0.551, 0.443	0.969
Age, year	0.004	−0.021, 0.029	0.745	−0.015	−0.035, 0.009	0.202
ACD, mm	0.175	−0.226, 0.575	0.390	−0.048	−0.415, 0.322	0.797
Km, D	−0.019	−0.163, 0.125	0.794	0.048	−0.092, 0.179	0.486
AL, mm	0.082	−0.025, 0.188	0.132	0.073	−0.063, 0.153	0.161
SE, D	0.075	0.020, 0.129	0.057	0.077	0.021, 0.124	0.003^*^
BCDVA, logMAR	1.056	0.514, 1.597	<0.001^*^	0.886	0.353, 1.462	0.002^*^

* *Significant at p <0.05. β =coefficient value. ACD, anterior chamber depth; AL, axial length; BCDVA, best corrected distance visual acuity; PS, posterior staphyloma; SE, spherical equivalent. Other type of staphyloma as the reference when comparing the different groups of PS associated with refractive error*.

### Factors Associated With Refractive Error in Different PS Types

In the univariate and multivariate analysis (Table 4), in eyes with wide macular staphyloma worse preoperative BCDVA was significantly associated with higher postoperative refractive errors (*P* < 0.05); however, there was no significant association between axial length (*P* < 0.05) and postoperative refractive errors. In eyes with narrow macular staphyloma, a longer axial length (*P* < 0.05) was significantly associated with higher postoperative refractive errors. In eyes with other types of posterior staphyloma, none of the examined risk factors was significantly correlated with postoperative refractive errors (all *P* > 0.05).

**Table 4:** Impact of risk factors on postoperative refractive errors in wide macular, narrow macular, and other types of posterior staphyloma.

**Variables**	**Univariate**			**Multivariate**		
	**β**	**95%CI**	***P***	**β**	**95%CI**	***P***
**Wide, macular (*****n*** **=** **52)**						
Age, year	0.012	−0.028, 0.052	0.552	−0.012	−0.053, 0.029	0.573
ACD, mm	0.129	−0.561, 0.818	0.709	−0.022	−0.690, 0.646	0.948
Km, D	0.028	−0.210, 0.266	0.814	−0.024	−0.259, 0.211	0.838
AL, mm	−0.022	−0.162, 0.118	0.752	−0.011	−0.181, 0.160	0.901
SE, D	0.056	−0.018, 0.130	0.133	0.072	−0.016, 0.160	0.104
BCDVA, logMAR	1.331	0.353, 2.309	0.009^*^	1.635	0.504, 2.766	0.006^*^
**Narrow, macular (*****n*** **=** **45)**						
Age, year	−0.023	−0.058, 0.011	0.180	−0.033	−0.067, 0.000	0.051
ACD, mm	0.140	−0.349, 0.629	0.568	−0.121	−0.612, 0.369	0.619
Km, D	0.001	−0.193, 0.191	0.990	0.117	−0.083, 0.318	0.243
AL, mm	0.202	0.040, 0.363	0.016^*^	0.197	0.020, 0.375	0.030^*^
SE, D	0.070	−0.017, 0.156	0.113	0.072	−0.014, 0.158	0.099
BCDVA, logMAR	0.573	−0.126, 1.271	0.105	0.447	−0.301, 1.195	0.234
**Others (*****n*** **=** **16)**						
Age, year	0.003	−0.039, 0.045	0.890	0.029	−0.050, 0.108	0.424
ACD, mm	−0.112	−1.117, 0.893	0.814	−0.602	−2.777, 1.574	0.547
Km, D	−0.129	−0.388, 0.129	0.301	−0.230	−0.645, 0.186	0.243
AL, mm	0.095	−0.229, 0.419	0.538	0.172	−0.251, 0.594	0.382
SE, D	−0.004	−0.114, 0.106	0.942	0.037	−0.162, 0.235	0.679
BCDVA, logMAR	0.401	−0.824, 1.625	0.494	0.060	−1.753, 1.873	0.942

* *Significant at P <0.05. β = coefficient value. ACD, anterior chamber depth; AL, axial length; BCDVA, best corrected distance visual acuity; PS, posterior staphyloma; SE, spherical equivalent*.

## Discussion

High myopia has a complex and variable course, and the associated mechanisms remain unclear (3, 19). PS is a common complication and one among other major causes of developing myopic maculopathy, which is identified as an outpouching of a circumscribed region of the posterior fundus prudent (16). Precise estimates of IOL power are critical to cataract surgery outcomes in patients with PS in highly myopic eyes. Axial length is the main risk factor affecting postoperative RE, in particular, in patients with high myopia (20). Using more advanced optical coherence interferometry instruments to measure axial length and revise the IOL power calculation formulas may help improve outcomes (21–23). Presently, IOL power prediction is more accurate than before; however, an unexpected significant RE (usually hyperopic) is not uncommon after cataract surgery for eyes with PS.

Except for axial length elongation in PS, a wide variation was observed in the morphological characteristics of eyeballs with PS (3). However, to our knowledge, there have been no studies on the association between different types of PS and RE after cataract surgery.

Advances in wide-field imaging have enabled the visualization of 200° of the retinal area, providing more detailed and accurate assessments of PS than those available previously (10, 12). Compared with conventional 50° fundus photography, wide-field imaging may detect the entire extent of PS, which is especially important for identifying the border of wide macular staphyloma. In our study, we used wide-field fundus imaging to detect different types of PS. In 113 highly myopic eyes with PS, the rates of wide, narrow, and other types of PS were 46.02, 39.82, and 14.16%, respectively. Shinohara et al. compared the rates of PS types in 117 eyes with retinoschisis and staphyloma, reporting 47.86, 48.72, and 3.42% of wide macular staphyloma, narrow macular staphyloma, and other types of PS, respectively (19). The reason for this discrepancy in findings may be the differences in study populations; the present participants were high myopia patients with PS who had undergone cataract surgery.

In our study, different types of PS were associated with RE. The development of staphyloma is usually accompanied various kinds of myopic lesions, such as CNV, macular epiretinal membranes and macular retinoschisis, which may influence the RE, therefore our research excluded these serious myopic retinopathies. There were significant differences in postoperative RE among wide macular staphyloma with narrow macular staphyloma and other types of PS in highly myopic eyes. Eyes with wide macular staphyloma have a higher risk of hyperopic RE after cataract surgery in highly myopic patients. In fact, the proposed subclassification of PS emerged as a functional factor that may play an essential role in predicting postoperative RE.

Our results have shown that highly myopic patients with wide macular staphyloma had lower corrected visual acuity than those with narrow macula and other types of staphylomas after cataract surgery (Table 1). Although there was no significant difference in BCDVA among patients with different PS before cataract surgery, we inferred that it may be partly affected by the clouded lens. The loss of BCDVA is closely related to the degree of myopic retinopathy. With the enlargement of myopic retinopathy, wide macular staphyloma may lead to a higher incidence of myopia-associated complications, such as diffuse chorioretinal atrophy, myopic choroidal neovascularization, and patchy chorioretinal pathological changes (24). The present findings were consistent with those of Ohno-Matsui, showing that the progression of visual impairment was significantly different among different types of PS in high myopia (10).

In highly myopic patients, there is a significant association between axial length elongation and increased RE after cataract surgery. In the present study, there was no significant difference in axial length among different types of PS (Table 1); however, the RE varied significantly among the different subclassifications after cataract surgery (Figure 2), which indicated that axial length may not be an accurate predictor of RE in highly myopic patients in clinical practice. This finding suggests that wide macular staphyloma may lead to a more hyperopic RE shift after cataract surgery (Table 2, Figure 2), which means that the type of PS might be a more effective predictor of postoperative RE in eyes with high myopia and PS.

Univariate and multivariate linear regression analyses further demonstrated that the presence of wide macular staphyloma was a significant predictor of postoperative RE (Table 3). The causal relationship between PS classification and postoperative RE warrants further study. Classifications of PS may interfere with accurate calculation of the RE in eyes with high myopia. First, axial length measurements are not accurate in myopic eyes with different types of PS, as the eye shapes are non-spherical and deformed due to PS (25). Axial length is the distance from the pre-surface of the central cornea to the fovea. When the eye is spherical, the diameter of the spherical globe is the same, and the axial length measurement is accurate. However, if the eye is irregularly deformed, the axial length is not a diameter, and the measured axial length is usually shorter than the actual axial length. Second, poor central fixation stability of the fovea caused by PS may affect refractive outcomes. In physical conditions, the magnitudes of central fixation movements of healthy eyes are small, while in eyes with myopic retinopathy, central fixation stability of the fovea is impaired and fluctuates greatly (26). Zhu et al. reported that poor fixation stability might have a positive relationship with RE after cataract surgery in eyes with high myopia (27). Wide macular staphyloma tends to be associated with more serious myopic retinopathy than is narrow macular staphyloma or other types of PS (12, 28, 29), which may account for the impairment of central fixation stability of the fovea. Third, a more advanced fundus status with wide macular staphyloma may affect the accuracy of axial length measurement, which would increase the refractive power of the eye. In our study, among eyes with wide macular staphyloma, there was no statistically significant association between axial length and postoperative refractive error; however, in eyes with narrow macular staphyloma, a statistically significant association between axial length and postoperative RE was detected (Table 4). Overall, these findings suggest that patients with wide macular staphyloma have larger RE than do other patients after cataract surgery; meanwhile, axial length is not the main factor affecting postoperative RE. However, as we just did the primary research on the relationship between wide macular staphyloma and RE after cataract surgery, the inner causal relationship between PS classification and postoperative RE warrants further study.

Our findings highlight that wide macular staphyloma may serve as an important source of postoperative RE in highly myopic cases. The present findings suggest that even highly myopic cases seem to present no significant difference in axial length, depending on the subclassification of PS evaluated by wide-field fundus imaging. The present findings are consistent with those of previous studies on eyes with high myopia and PS (30), showing that refractive outcomes vary significantly among different types of PS after cataract surgery. In addition, a significant correlation between posterior staphyloma classification and postoperative RE was observed. Wide macular staphyloma type was more strongly associated with postoperative RE than was narrow macular and other types staphyloma types; this finding suggests that, to a certain extent, it may serve as a biomarker for predicting higher RE after cataract surgery.

There are some limitations to the present study. First, the number of PS types was relatively small, which partly limited the statistical power of the analysis. Second, Ohno-Matsui's classification of PS involves six types (wide macular staphyloma, narrow macular staphyloma, inferior staphyloma, peripapillary staphyloma, and nasal staphyloma, and types not otherwise specified). We combined the latter four types into the “other” types of PS (14.16% of PS in our study); however, it remains difficult to differentiate refractive outcomes of these four types. Lastly, except posterior staphyloma, dome shaped macula, myopic choroidal neovascularization, and macular atrophy are the very common complications of high myopia, which might be potential predictive biomarkers for visual acuity after cataract surgery in highly myopic eyes. In our future study, we aim to include larger samples of PS and compare the rates of myopic retinal complications among the different types, such as dome-shaped macula, myopic choroidal neovascularization, or macular atrophy, to examine factors associated with RE after cataract surgery and we will research and comment on the types of myopic retinopathy among different kinds of staphyloma in this context. Besides, the depth of PS detected by wide-field OCT may have an impact on postoperative RE, which deserves further study.

In conclusion, this study classified the PS of highly myopic patients with cataract surgery according to their morphologic characteristics, identified using wide-field fundus imaging, and examined refractive outcomes among the three types of PS. The subclassification of staphyloma may be a useful biomarker for future prediction of postoperative RE in highly myopic eyes; wide macular staphyloma might have a detrimental effect on the accuracy of postoperative refractive outcome prediction. Therefore, PS classification should be considered in clinical practice, specifically, for highly myopic patients due to undergo cataract surgery with wide macular staphyloma.

## Data Availability Statement

The original contributions presented in the study are included in the article/supplementary material, further inquiries can be directed to the corresponding author.

## Ethics Statement

The studies involving human participants were reviewed and approved by the Clinical Research Ethical Committee of Shanghai Tenth People's Hospital affiliated with Tongji University. The patients/participants provided their written informed consent to participate in this study.

## Author Contributions

JW: writing the original draft and data analysis. RW: data analysis and editing. CL, YZ, and ZJ: data collection. FL: supervision and editing. All authors contributed to the article and approved the submitted version.

## Funding

This study was supported by the Clinical Research and Cultivation Project of Shanghai Municipal Hospital (SHDC12019X30) and Science and Technology Commission of Shanghai Municipality (20142203200).

## Conflict of Interest

The authors declare that the research was conducted in the absence of any commercial or financial relationships that could be construed as a potential conflict of interest.

## Publisher's Note

All claims expressed in this article are solely those of the authors and do not necessarily represent those of their affiliated organizations, or those of the publisher, the editors and the reviewers. Any product that may be evaluated in this article, or claim that may be made by its manufacturer, is not guaranteed or endorsed by the publisher.
